# Supplementary material for: Comprehensive Mosquito Wing Image Repository for Advancing Research on Geometric Morphometric- and AI-Based Identification
Source: Sci Data. 2025 Apr 29;12:715. doi: 10.1038/s41597-025-05043-3 (PMC12041405; doi:10.1038/s41597-025-05043-3)
Supplement: Supplementary file 1 — Supplementary Tables [file 41597_2025_5043_MOESM1_ESM.pdf]

Supplementary Table 1

Table 1: Distribution of specimens and images separated by taxonomic levels.

1. Taxonomic Level	2. Taxonomic Level	3. Taxonomic Level	4. Taxonomic Level	5. Taxonomic Level	Number of Images	Number of Specimens
Family	*Genus*	*Species, Species group, Species complex or Species pairs*	*Species*	*Subspecies or biotypes*		
Culicidae	*Aedes*	-	*aegypti*	-	2916	1300
Culicidae	*Aedes*	-	*albopictus*	-	1344	558
Culicidae	*Aedes*	-	*amesii*	-	2	1
Culicidae	*Aedes*	-	*caspius*	-	82	82
Culicidae	*Aedes*	-	*cataphylla*	-	148	76
Culicidae	*Aedes*	-	*cyprus*	-	4	2
Culicidae	*Aedes*	-	*geniculatus*	-	24	24
Culicidae	*Aedes*	-	*imprimensis*	-	1	1
Culicidae	*Aedes*	-	*intrudens*	-	17	9
Culicidae	*Aedes*	-	*japonicus*	japonicus	1772	689
Culicidae	*Aedes*	-	*koreicus*	-	1092	350
Culicidae	*Aedes*	-	*ostentatio*	-	19	13
Culicidae	*Aedes*	-	*pulchritarsis*	-	2	2
Culicidae	*Aedes*	-	*rossicus*	-	14	14
Culicidae	*Aedes*	-	*rusticus*	-	229	229
Culicidae	*Aedes*	-	*sticticus*	-	233	232
Culicidae	*Aedes*	-	*thailandensis*	-	2	1
Culicidae	*Aedes*	-	*vexans*	-	455	450
Culicidae	*Aedes*	-	*vittatus*	-	2	2
Culicidae	*Aedes*	*annulipes-*group	-	-	354	214
Culicidae	*Aedes*	*cinereus-geminus-*pair	-	-	339	288
Culicidae	*Aedes*	*communis-punctor-*pair	-	-	580	492
Culicidae	*Anopheles*	-	*aconitus*	-	1	1
Culicidae	*Anopheles*	-	*daciae*	-	18	9
Culicidae	*Anopheles*	-	*epiroticus*	-	6	3
Culicidae	*Anopheles*	-	*hyrcanus*	-	20	20
Culicidae	*Anopheles*	-	*messeae*	-	31	16

Culicidae	*Anopheles*	-	*moucheti*	-	63	33
Culicidae	*Anopheles*	-	*paludis*	-	65	33
Culicidae	*Anopheles*	-	*plumbeus*	-	51	47
Culicidae	*Anopheles*	-	*sinensis*	-	19	12
Culicidae	*Anopheles*	-	*stephensi*	-	251	132
Culicidae	*Anopheles*	*claviger-petragnani-*pair	-	-	136	118
Culicidae	*Anopheles*	*coustani-*group	-	-	80	41
Culicidae	*Anopheles*	*gambiae* s.l.	-	-	42	22
Culicidae	*Anopheles*	*maculipennis* s.l.	-	-	50	39
Culicidae	*Anopheles*	*maculipennis* s.l.	*atroparvus*	-	57	57
Culicidae	*Anopheles*	*maculipennis* s.l.	*daciae*	-	62	62
Culicidae	*Anopheles*	*maculipennis* s.l.	*maculipennis*	-	98	98
Culicidae	*Anopheles*	*maculipennis* s.l.	*messeae*	-	376	376
Culicidae	*Anopheles*	*nili* s.l.	-	-	29	15
Culicidae	*Anopheles*	*tessellatus*	-	-	2	1
Culicidae	*Armigeres*	-	*durhami*	-	1	1
Culicidae	*Armigeres*	-	*jugraensis*	-	1	1
Culicidae	*Armigeres*	-	*moultoni*	-	2	1
Culicidae	*Armigeres*	-	*subalbatus*	-	75	46
Culicidae	*Coquillettidia*	-	*buxtoni*	-	2	2
Culicidae	*Coquillettidia*	-	*crassipes*	-	63	34
Culicidae	*Coquillettidia*	-	*richiardii*	-	104	104
Culicidae	*Coquillettidia*	-	*subalbatus*	-	2	1
Culicidae	*Culex*	-	*bitaeniorhynchus*	-	8	4
Culicidae	*Culex*	-	*brevipalpis*	-	8	5
Culicidae	*Culex*	-	*fuscocephala*	-	38	23
Culicidae	*Culex*	-	*gelidus*	-	47	27
Culicidae	*Culex*	-	*modestus*	-	146	111
Culicidae	*Culex*	-	*nigropunctatus*	-	9	6
Culicidae	*Culex*	-	*orientalis*	-	4	3
Culicidae	*Culex*	*pipiens* s.l.-*torrentium-*pair	-	-	2684	1885

Culicidae	*Culex*	*pipiens* s.l.-*torrentium*-pair	*pipiens*	biotype molestus	353	195
Culicidae	*Culex*	*pipiens* s.l.-*torrentium*-pair	*pipiens*	biotype pipiens	1913	978
Culicidae	*Culex*	*pipiens* s.l.-*torrentium*-pair	*quinquefasciatus*	-	226	151
Culicidae	*Culex*	*pipiens* s.l.-*torrentium*-pair	*torrentium*	-	462	240
Culicidae	*Culex*	*territans-hortensis*-pair	-	-	22	22
Culicidae	*Culex*	*vishnui*-group	-	-	204	124
Culicidae	*Culex*	*vishnui*-group	*pseudovishnui*	-	18	9
Culicidae	*Culex*	*vishnui*-group	*tritaeniorhynchus*	-	385	195
Culicidae	*Culex*	*vishnui*-group	*vishnui*	-	8	4
Culicidae	*Culiseta*	-	*alaskaensis*	-	2	1
Culicidae	*Culiseta*	*annulata-subochrea*-pair	-	-	50	50
Culicidae	*Culiseta*	*morsitans-fumipennis*-pair	-	-	168	107
Culicidae	*Mansonia*	-	*indiana*	-	3	2
Culicidae	*Mansonia*	-	*uniformis*	-	6	4
Culicidae	*Toxorhynchites*	*splendens*	-	-	1	1
Culicidae	*Uranotaenia*	-	*unguiculata*	-	1	1

Supplementary Table 2

Table 2: Description of meta parameters annotated to each image in the dataset

Parameter	Description	Format	Example
1. Taxonomic Level	Family	string	Culicidae
2. Taxonomic Level	Genus	string	*Culex*
3. Taxonomic Level	Species Group, Species complex or Species pairs	string	*pipiens* s.l.-*torrentium*-pair
4. Taxonomic Level	Species	string	*pipiens*
5. Taxonomic level	Subspecies and biotypes	string	biotype *pipiens*
Capture Location	The location in which the sample was collected	string	Hamburg, Germany
Capture location - LAT	Latitude in which the sample was collected.	float	51.445989
Capture location - LONG	Longitude in which the sample was collected.	float	13.519128
Capture method	The method used to capture the specimen	string	Ovitrap
Damaged Wing	If the wing was damaged to the extent that key morphological characteristics were missing	boolean	True
Date Database Addition	The date on which the image was added to the database	DateTime	28.08.2024
Date of Sample Collection	The day on which the sample was collected	DateTime	28.08.2024
Device	Microscope and camera used for image capture	string	Olympus SZ81 + Olympus DP23
File Name	Name given to File according to the protocol described in Data Records	string	culex_aida_f_r_3075170417c4.tif
Files	The full path of the image in the database	string	Culicidae/culex/culex_aida_f_r_3075170417c4.tif
Image ID	Unique Identifier attributed to each image by MD5 encryption of the original file path	string	aa79004529fb

Method of Identification	Method used to identify the species of the specimen	string	ITS2 gene barcoding
Observer	The person responsible for image capture	string	K. Nolte
Organisation	The organisation responsible for Project	string	Bernhard Nocht Institute for Tropical Medicine
Project	Project Identifier	string	AIDA
Project Description	Short description of the project's objective	string	A proof-of-concept study to analyse the potential of a CNN to identify mosquito species based on wing images.
Publication DOI	The referenced DOI if the study was already published	string	10.1128/spectrum.00128-24
Sex	Sex of the specimen	character	F: female<br>M: male
Specimen ID	A unique identifier given to each mosquito specimen in the dataset	integer	4045
Wing side	As seen from the dorsal aspect of the mosquito, with its head oriented away	character	R: right<br>L: left
